# Soil Nutrient Detection for Precision Agriculture Using Handheld Laser-Induced Breakdown Spectroscopy (LIBS) and Multivariate Regression Methods (PLSR, Lasso and GPR)

**DOI:** 10.3390/s20020418

**Published:** 2020-01-11

**Authors:** Alexander Erler, Daniel Riebe, Toralf Beitz, Hans-Gerd Löhmannsröben, Robin Gebbers

**Affiliations:** 1 Physical Chemistry, University of Potsdam, Karl-Liebknecht-Str. 24-25, 14476 Potsdam, Germany; aerler@uni-potsdam.de (A.E.); riebe@uni-potsdam.de (D.R.); beitz@uni-potsdam.de (T.B.); 2 Leibniz Institute for Agricultural Engineering and Bioeconomy (ATB), Max-Eyth-Allee 100, 14469 Potsdam, Germany; rgebbers@atb-potsdam.de

**Keywords:** LIBS, lasso, PLS regression, gaussian processes, soil, precision agriculture, nutrients

## Abstract

Precision agriculture (PA) strongly relies on spatially differentiated sensor information. Handheld instruments based on laser-induced breakdown spectroscopy (LIBS) are a promising sensor technique for the in-field determination of various soil parameters. In this work, the potential of handheld LIBS for the determination of the total mass fractions of the major nutrients Ca, K, Mg, N, P and the trace nutrients Mn, Fe was evaluated. Additionally, other soil parameters, such as humus content, soil pH value and plant available P content, were determined. Since the quantification of nutrients by LIBS depends strongly on the soil matrix, various multivariate regression methods were used for calibration and prediction. These include partial least squares regression (PLSR), least absolute shrinkage and selection operator regression (Lasso), and Gaussian process regression (GPR). The best prediction results were obtained for Ca, K, Mg and Fe. The coefficients of determination obtained for other nutrients were smaller. This is due to much lower concentrations in the case of Mn, while the low number of lines and very weak intensities are the reason for the deviation of N and P. Soil parameters that are not directly related to one element, such as pH, could also be predicted. Lasso and GPR yielded slightly better results than PLSR. Additionally, several methods of data pretreatment were investigated.

## 1. Introduction

Soils usually exhibit an inhomogeneous distribution of chemical, physical and biological soil properties. On agricultural land, this creates spatial variations of the qualities relevant for soil fertility and related management measures such as tillage, seeding, and fertilization. Chemical soil fertility parameters include the total contents of the main nutrients, such as potassium, magnesium, calcium, nitrogen and phosphorus, the total contents of the trace nutrients such as iron and manganese, the plant availability of these nutrients, the humus content and the soil pH value. The usual, uniform fertilization of the fields can lead to partial over-or underdosing. Overdosing can lead to exposure of surface and groundwater. Underdosing can result in an under-supply with nutrients, and thus to losses in yield and quality at the site. The concept of site-specific plant production (precision agriculture) can remedy this situation. The basic idea is to record the spatial variations within a field and to react to them in an appropriate way. Precision agriculture therefore requires spatially differentiated information which needs to be obtained in a cost and time efficient manner [1]. Instead of time-consuming and expensive soil sampling with consecutive laboratory analysis, in- or ex-situ soil sensors can be used. However, only a few sensor technologies, such as geoelectrical, potentiometric pH, gamma-ray and spectral-optical sensors, are currently used [2,3,4].

A promising method, which is potentially well suited for the in-field determination of total contents of elements in soils, is laser-induced breakdown spectroscopy (LIBS). LIBS is an optical emission spectroscopy technique [5,6,7,8,9]. An intense pulse of laser radiation, typically of nanosecond duration, is focused onto the sample, where it ablates material from the surface and creates a microplasma. The plasma, in turn, excites atoms and atomic ions which emit radiation specific to the elemental composition of the sample. The technique can thus be used for simultaneous multi-element analysis. In comparison to X-ray fluorescence (XRF), which also gains interest as method for rapid soil analysis [10], the whole range of elements, including the light elements, is accessible by LIBS. This allows the direct (in situ) analysis of macro and trace nutrients because no or only minimal sample preparation is necessary. Additionally, LIBS measurements are much faster than XRF measurements. The laser beam is usually focused to a spot with a diameter between 10 and 500 µm in order to reach the threshold for plasma generation. Therefore, the soil heterogeneity at the micrometer scale has to be considered for obtaining representative results. With today’s technology robust and portable LIBS systems can be build. These characteristics of LIBS make it a suitable tool for cost-effective and fast on-site analyses, compared to the practice of laboratory analyses, and make LIBS appear particularly well suited for the spatially resolved soil analysis of agricultural fields.

Ablation and plasma excitation are both highly complex phenomena. Since the interaction of the laser with the sample is influenced by the sample composition, a matrix-dependence of the signal response is observed. These matrix effects lead to a non-linear relation between the elemental content and the intensity of the spectral line. Matrix effects can have different sources, including the laser-to-sample coupling, collisional interactions within the plasma, or the plasma temperature, which influences the ratio of neutral and ionized species. These matrix effects and spectral interferences were already investigated for Pb determined in different types of soil [11]. The study demonstrated that spectral interferences from main components (Ca, Fe, Ti, Mg) and trace components (Mn, Nb, Zr) were a reason for significant differences between the univariate calibration models. In another work, the influence of the moisture content, the compressive force for sample pelleting and the total content of easily ionized elements on the ionization equilibrium were characterized [12].

Depending on the calibration effort, the elements can be determined qualitatively or quantitatively. While univariate calibration suffers from the matrix effects and corresponding calibration models often cannot be transferred to other soil types, multivariate analysis of whole spectra intrinsically takes matrix effects into account. A common, robust and reliable multivariate method used for calibrating LIBS for soil analysis is partial least squares regression (PLSR). PLSR is a method of dimensional reduction, which first identifies a new reduced set of features that are linear combinations of the original features, and then fits a linear model via least squares using these new features. However, this and similar methods have two limitations. First, all spectral channels are used, although many channels only contribute noise. Second, a spectrum can consist of several hundred lines. It is interesting to know which lines contribute to the analysis of the element investigated. Methods which have the ability to reduce the number of input variables (spectral channels) and to correlate a smaller number of input variables are better suited for model interpretation [13]. One method enabling this reduction is the least absolute shrinkage and selection operator (Lasso) [14]. Lasso is very similar to least squares regression, except that it includes a penalty term of the 𝓁_1_ norm. This penalty term limits the absolute sum of all coefficients in the linear regression equation and shrinks the coefficient estimates generally towards zero. Furthermore, the penalty term of the 𝓁_1_ norm has the property of forcing most coefficients to zero. This often leads to regression functions with drastically reduced sets of predictors. Multivariate analysis by PLSR and Lasso was already evaluated for LIBS investigations of geological samples [15,16,17]. Both methods yield similar results. However, Lasso enables a better interpretation. In addition to PLSR and Lasso, a broad spectrum of machine learning methods such as artificial neural networks (ANN) and support vector machines (SVR) have been applied to the quantification and classification of geological samples investigated by LIBS. A method seldom used with LIBS is Gaussian process regression (GPR) [18]. In contrast to PLSR and Lasso, GPR is a non-parametric method. It is a local regression approach, which uses a kernel for weighting neighboring observations in the estimation. Compared to other kernel methods, such as splines and support vector machines, GPR is slower but yields properly tuned probabilistic outputs and is sometimes more robust and flexible.

While publications on large-scale field mapping by LIBS are scarce [19], the detection of soil nutrients in the laboratory has already been demonstrated in a series of publications [20]. For example, in a study by Guo et al., 13 different elements in 17 different standard soils were determined by LIBS applying PLSR and SVR [21]. Single pulse and double pulse LIBS were compared at the determination of K, Ca, Mg, Fe, Na and Mn in 63 soil samples applying PLSR and SVR [22]. Further work describes the application of the laser ablation-LIBS method for the determination of Fe, Mn, Mg, Ca, Na and K in soil samples [23], the univariate analysis of Ca, Mg, P, Fe and Na [24], and the analysis of Ba, Co, Cu, Mn, Ni, V and Zn applying a multilayer perceptron ANN [25]. A special focus was the determination of the organic, inorganic and total carbon content for characterizing the soils in agriculture and the carbon sequestration potential. These investigations started with univariate analysis of the total carbon content using the two C lines at 193 nm [26,27] and 247.9 nm [28]. Later, multivariate methods such as PLSR [29,30,31], Lasso and multivariate regression with covariance estimation [15,16,17,18,19,20,21,22,23,24,25,26,27,28,29,30,31,32] were applied, which also allowed the differentiation of inorganic and organic C. Multivariate approaches were also used to determine further soil parameters such as soil pH [33].

While most work on LIBS was performed with laboratory instrumentation, a demonstration of the feasibility of using mobile or handheld equipment is necessary for a future in-field application of LIBS. First field-portable instruments were introduced and characterized in 2001 [34] and 2005 [35]. With the commercial availability of handheld LIBS instruments utilizing microchip lasers with pulse energies around 6 mJ, and broadband spectrometers covering a spectral range from 190 to 950 nm, geological and environmental applications became possible. In these works, the applicability of the handheld instruments for geochemical fingerprinting [36], calibration curves of Cu and Al alloys [37] and geological discrimination of, e.g., meteors and fakes [38,39] were investigated. Further applications are described in two reviews on portable spectroscopy [40,41].

This work had three objectives. First, the potential of a handheld instrument for the determination of a broad range of major (Ca, Mg, K, P and N), and minor (Mn and Fe) nutrients was investigated. Furthermore, the capability of detecting non-elemental soil parameters, such as the humus content and the soil pH, was assessed. A first screening of the potential of measuring the plant available content of P was carried out. Second, three different multivariate regression methods for the nutrient determination in soils using LIBS were evaluated. The methods are PLSR, Lasso and GPR. Third, different methods of data preparation with the aim of improving the results of multivariate regressions were investigated. Even though it is known that data pre-processing can have a large impact on the calibration performance, there is no general consensus for a unified procedure.

## 2. Materials and Methods

### 2.1. Soil Sampling, Reference Analytics and Sample Preparation

The samples were taken from the topsoil (0 to 30 cm) of two agricultural fields near Wilmersdorf (Germany, 53°06’ N, 13°54’ E). The main parent material of the soil consists of calcareous glacial till with a cover of non-stratified sand and stones. The soil texture varies between sand, loamy sand and sandy loam in the topsoil. In total, 68 and 69 samples were taken from the fields. The sampling procedure and sample selection were described earlier [42]. All 137 samples were characterized by reference analytics. While the total mass fractions of the elements (Ca, K, Mg, N, P, Fe, Mn, Al) were determined by wet digestion and ICP-OES, the mass fractions of P available for plants were determined after double lactate extraction, the humus content by the determination of the organic carbon and the pH value by suspension of the soil in aqueous CaCl_2_ solution and pH electrode measurement.

Soil pellets were produced by taking 3 g of soil sample and mixing with 90 µL water to establish a standardized moisture. Then, the soil samples were homogenized using a ball mill (MM 400, Retsch, Hahn, Germany) and pressed to pellets at 50 kN without applying binding agents (TP 40, Herzog Maschinenfabrik, Osnabrück, Germany).

### 2.2. LIBS Apparatus

The pellets were measured using a LIBS handheld instrument (Z-300, SciAps, WBN, MA). The spectrometer has a detection range of 190–950 nm. The integrated laser emits radiation at a wavelength of 1064 nm, with a repetition rate of 10 or 50 Hz and ca. 7.5 mJ pulse energy. For the measurements described in this paper, a repetition rate of 10 Hz was used. Additionally, the device provides an Ar gas purge to remove ambient air and enhance the LIBS signals. The measurements consisted of 64 shots in an 8 × 8 grid of approximately 1 mm² area size on the surface of the pellet. The measurements were repeated three times per pellet in order to obtain a representative (averaged) spectrum of the sample.

### 2.3. Preprocessing of Data

**Variance reduction.** The LIBS spectra show relatively large fluctuations of their intensities. The reasons for these variations are, e.g., the normal stochastic plasma fluctuations and variations due to micro inhomogeneities in the ground soils pressed to pellets. One possibility for the reduction of variations is averaging. Another way is variance reduction based on principle component analysis (PCA) proposed by Pořizka et al., [43]. In this method, the Euclidean distance of a data point to the center of the principal component (PC) space constructed by the first three principal components is determined. The method removes a predetermined percentage of spectra with the largest Euclidean distances before averaging the remaining spectra of one sample point. In this approach, the coefficients of determination of PLSR were compared to assess the effect of the removal of none, 5%, 20% and 50% of the spectra with the largest distances to the center of gravity in PCA. Upon the removal of 5%, 20% or 50% of the spectra, all remaining spectra of one data point were averaged.

**Background correction and normalization.** A top-hat filter [44] with a structure element length of 20 data points was used for background correction. Standard normal variate (SNV) normalization was used [45].

**Data reduction.** In a field measurement campaign, a huge amount of data is potentially generated. LIBS spectra contain a large number of data points that may not all be relevant. Besides creating unnecessary computational burden, irrelevant data can negatively affect calibration and prediction. The approach for data reduction used here is based on background correction followed by integration of all lines in the spectrum. Background correction and integration of spectra were performed in the open source software OpenMS [46] which was developed for mass spectrometric data processing. One advantage of this software is the option of batch processing that allows the automatic processing of large amounts of data. The top-hat filter was used for background correction. The integration tool includes a threshold parameter which determines the signal-to-noise ratio at which the lines are integrated, and thus determines the number of lines selected and the extent of data reduction. While a high threshold can decrease the number of lines to the most intense lines, a low threshold can include the noise of the baseline. Therefore, five different thresholds were selected, which consider both extreme cases and three levels in between.

### 2.4. Data Analysis by Multivariate Methods

Three multivariate methods were used for obtaining calibration models. PLSR is widely applied in the LIBS community and can be regarded as a reference method. Lasso regression is a shrinkage method which constrains the coefficient estimates and shrinks coefficient estimates that do not significantly contribute to the correlation towards zero. This enables a robust regression and a simplified interpretation of the coefficients. In this work, the number of coefficients was always reduced to the minimum number possible (Min) and the number necessary for an error one standard deviation above that minimum (1SE). GPR, also known as kriging in geostatistics, is a method rarely applied in LIBS [47]. GPR models are nonparametric kernel-based probabilistic models.

All methods were implemented in Matlab (Version 2019a, MathWorks, Natick, MA, USA). PLSR was carried out with *plsregress* and GPR was based on *fitrgp*. Both functions are included in Matlab’s Statistics and Machine Learning Toolbox. Lasso regression was performed with the *glmnet* function, which is part of a package provided by J. Friedman et al. [48]. Different validation procedures were tested, namely 10-fold cross-validation, random splitting of the 137 samples into a 50% training and a 50% test data set (*cvpartition* function in matlab) as well as using data of the first field as training data (50%) and data of the second field as test data (50%).

## 3. Results

**Figure 1:**
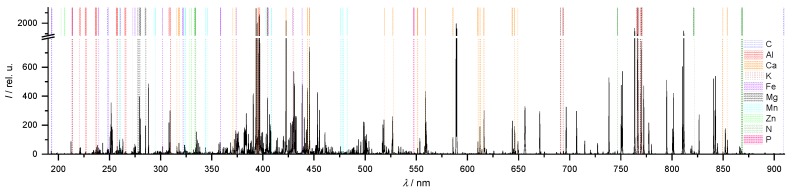
Representative laser-induced breakdown spectroscopy (LIBS) spectrum of a soil sample of the field near Wilmersdorf, lines of the elements investigated are marked by colored lines (labels on the right).

The focus of this work was the characterization of the potential of a handheld LIBS instrument for the determination of a broad range of major (Ca, Mg, K, P and N), and minor (Mn and Fe) nutrients in soils. These elements are among the most important nutrients for plants in agriculture. A typical LIBS spectrum of the soils recorded with the handheld instrument is displayed in Figure 1. The information of this spectrum is dense and its structure is complex. Important lines of the elements investigated are marked by colored lines. The emissions are found within the whole spectral range of the handheld spectrometer between 190 and 950 nm. The lines of all elements in the spectrum except N and P appear in high intensities.

The most intense lines of the observed nutrients and the average mass fractions of the nutrients obtained by reference analytics (ICP-OES) are summarized in Table 1. The mass fractions of all nutrients cover almost two orders of magnitude. This represents a challenge for an analytical investigation. For the evaluation of the handheld instrument, the variation of environmental parameters, such as moisture, grain size distribution (texture), and general heterogeneity of the soil, was reduced by sample pretreatment. This included drying, grinding, homogenizing and pressing the soil into pellets.

Though univariate calibration can be applied successfully to samples from a small geographic region, e.g., from one field, multivariate calibration methods consider matrix effects to a greater extent, often provide better and more generalized calibration models as well as a better prediction of unknown soil samples. In this work, three different multivariate methods, namely PLSR, Lasso and GPR, were characterized and compared. The regression models were validated in three different ways:

1. 10-fold cross validation as a general standard;
2. statistical splitting of the data set in 50% training data and 50% test data for comparison to the third validation scheme;
3. data from field 1 for training and data from field 2 for testing (splitting in 50% training and 50% test data).

The latter validation scheme is considered a real-world scenario and gives an indication for the generalization of the calibration model and its possible application on unknown fields.

### 3.1. Calcium

**Figure 2:**
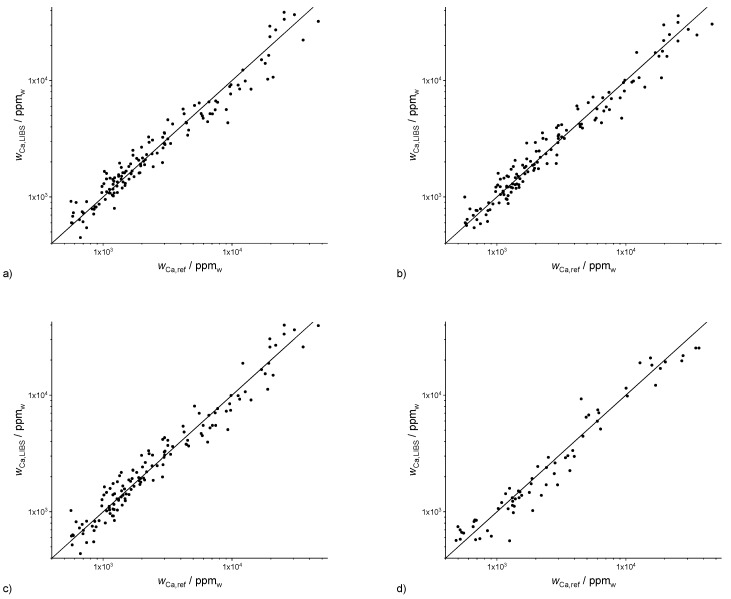
Results of 10-fold cross validation of Ca data for different multivariate methods (**a**) Lasso regression (R^2^ (log) = 0.85), (**b**) Gaussian process regression (GPR) (R^2^ (log) = 0.89), (**c**) partial least squares regression (PLSR) (seven components, R^2^ (log) = 0.87), and (**d**) PLSR of second field (six components, R^2^ (log) = 0.90).

The best overall results were obtained for calcium, which had the third largest average mass fraction behind Fe and Al (see Table 1). Some validation results of the three multivariate regression methods and the different validation schemes are shown in Figure 2. A more detailed summary is given in Table 2 along with the calibration performance for the other nutrients. 10-fold cross-validation of the three multivariate methods yields roughly similar results. PLSR is the most common method and can be regarded as the standard. Lasso and GPR are more rarely applied methods, which are compared with this standard. The coefficients of determination (R^2^) and root mean squared errors of prediction (RMSEP) are used as quantitative measures for the comparison of the three methods. Due to the wide range of Ca mass fractions of over two orders of magnitude, multivariate regressions were performed with both logarithmic (only for Ca) and non-logarithmic mass fractions. Consequently, R^2^ and RMSEP (Table S1 in Supplementary Material) are reported for both types of regression. Ten-fold cross-validation of PLSR of nearly 137 spectra yields good figures of merit (Figure 2c), i.e., in the case of logarithmic (R^2^ (Ca, PLSR) = 0.87) and non-logarithmic mass fractions (R^2^ (Ca, PLSR) = 0.86). The corresponding coefficients of determination obtained for Lasso were slightly worse (R^2^ (log, Ca, Lasso) = 0.85 and R^2^ (non-log, Ca, Lasso) = 0.84), since Lasso radically reduces the number of predictors (Figure 2a). GPR as a non-parametric method was the best method for the determination of Ca mass fractions (R^2^ (log, Ca, GPR) = 0.89, R^2^ (non-log, Ca, GPR) = 0.83) (Figure 2b).

Scenario 2 is closely related to validation scheme 3, but uses a randomly chosen selection of the samples into a split data set using 50% of the spectra for training and 50% of the spectra for validation. PLSR for the test data yields a good prediction, R^2^ (log, Ca, PLSR) = 0.89 (5 components, correlation not shown), which is similar to 10-fold cross validation.

A possible real-world scenario is using the samples of field 1 for calibration and applying this calibration to the prediction of the soil samples of another field (field 2). A successful application would reduce the calibration effort for extending the application of LIBS to further fields which would facilitate the adaption of the method. This transfer is very challenging due to the strong matrix effects encountered in soils. The application of the calibration to the test data of field 2 (Figure 2d) shows a surprisingly good prediction with R^2^ (Ca, PLSR) = 0.90, which allows a relatively precise estimation of Ca contents. These coefficients of determination are very similar to the corresponding values of the split data set obtained by random selection of sample points. This is an indication of similar chemical and physical soil properties (matrix effects) of the second field.

### 3.2. Magnesium and Potassium

**Figure 3:**
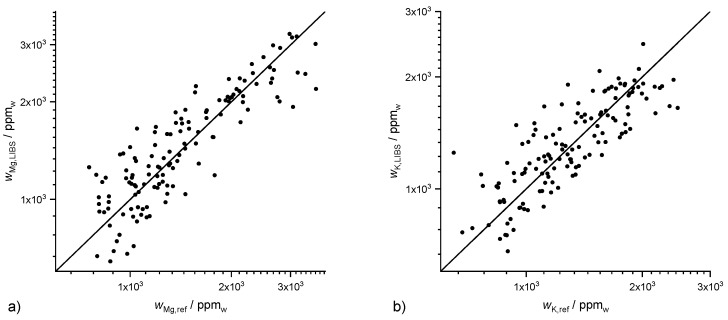
Results of 10-fold PLSR cross validation for (**a**) Mg with R^2^ (Mg, PLSR) = 0.79, and (**b**) K with R^2^ (K, PLSR) = 0.64.

While the averaged Ca mass fraction in the soils investigated is 4950 ppm, the averaged mass fractions of Mg and K are 1450 ppm and 1280 ppm and therefore significantly lower. This corresponds to lower line intensities in the LIBS spectrum (Figure 1). The coefficients of determination of the PLSR (10-fold CV) is R^2^ (Mg, PLSR) = 0.79 for Mg (Figure 3), which is lower than the Ca value. The value of R^2^ (K, PLSR) = 0.64 for K is even lower. The scattering of the data points increases for K but allows a rough estimate. Beside the lower mass fractions compared to Ca, the decreased predictive power in the case of Mg and K can also be attributed to the lower number of lines (N(Mg) = 5 and N(K) = 5 vs. N(Ca) = 23) observed in the LIBS spectrum. Furthermore, the two strong K lines at 766.4 nm and 769.9 nm are influenced by self-absorption, which makes a linear regression more difficult. In addition to these reasons, the dynamic range of the mass fractions of Mg and K is less than one order-of-magnitude and therefore much smaller than the corresponding mass fraction range of Ca, which covers two orders-of-magnitude.

The coefficients of determination of the three multivariate methods (10-fold CV) are R^2^ (Mg, PLSR) = 0.79 (Figure 3), R^2^ (Mg, Lasso) = 0.75 and R^2^ (Mg, GPR) = 0.78 for Mg as well as with R^2^ (K, PLSR) = 0.64, R^2^ (K, Lasso) = 0.65 and R^2^ (K, GPR) = 0.66 for K and thus quite similar. While PLSR yields the best prediction for Mg followed by GPR and Lasso, GPR is best for K followed by Lasso and PLSR. However, the differences between the results of the three methods are negligible.

### 3.3. Nitrogen and Phosphorus

**Figure 4:**
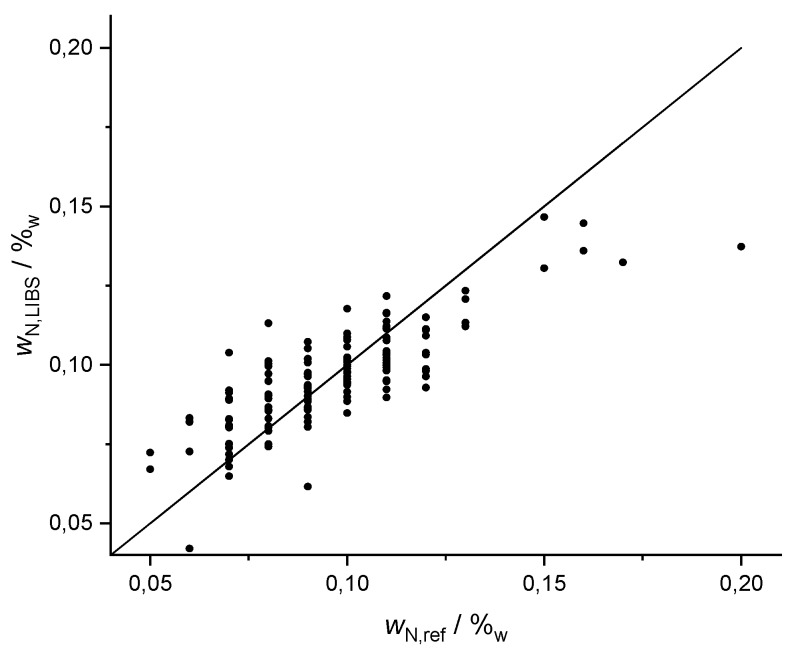
Results of 10-fold Lasso cross validation for nitrogen, R^2^ (N, Lasso) = 0.65, reference data of nitrogen is coarsely resolved (in classes of Δ 0.01%).

Nitrogen and phosphorus are two very important nutrients. Their average contents in the Wilmersdorf fields are relatively low compared to other elements, namely 917 ppm for nitrogen and 372 ppm for phosphorus. Due to these low mass fractions and the low line strengths of both elements, only a few weak lines for both elements could be assigned, three for nitrogen and two for phosphorus. However, it cannot be excluded that these lines are superimposed with signals from other elements, due to the many lines in the spectrum and the limited resolution (>0.1 nm) of the spectrometer. Though a robust prediction should not be expected with these weak lines, Lasso yielded good predictions for N with R^2^ (N, Lasso) = 0.65 (Figure 4) and a qualitative correlation between predicted and observed P values with R^2^ (P, Lasso) = 0.21. Similar coefficients of determination were obtained with PLSR: R^2^ (N, PLSR) = 0.51 and R^2^ (P, PLSR) = 0.14 as well as GPR with R^2^ (N, GPR) = 0.51 and R^2^ (P, GPR) = 0.28. The prediction results of the three methods were similar.

### 3.4. Minor Nutrients

**Figure 5:**
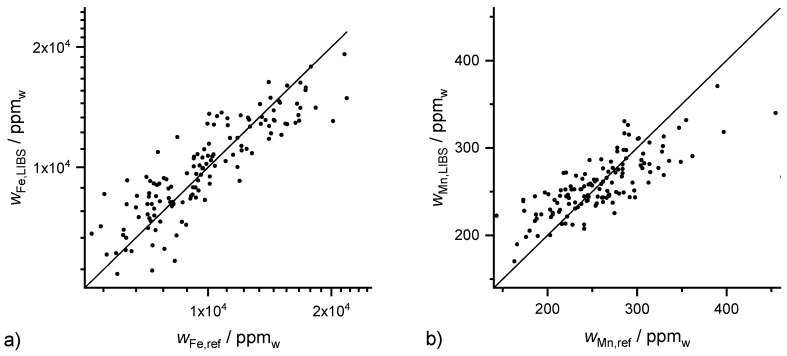
Results of 10-fold Lasso cross validation for (**a**) Fe with R^2^ (Fe, Lasso) = 0.76 and (**b**) Mn with R^2^ (Mn, Lasso) = 0.55.

The minor nutrients Mn and Fe were also investigated. Their average mass fractions of 10,400 ppm for Fe and 249 ppm for Mn vary strongly between both elements. The highest coefficients of deter-mination are obtained for Fe with R^2^ (Fe, Lasso) = 0.76 (Figure 5a) followed by Mn with R^2^ (Mn, Lasso) = 0.55 (Figure 5b). This ranking corresponds to the order of the mass fractions. In addition to the low mass fraction of Mn, the mass fraction range was also very narrow, e.g., compared to Ca. This is the reason for the decreasing coefficient of determination. The coefficient of determination obtained for Fe by PLSR was R^2^ (Fe, PLSR) = 0.77 and by GPR was R^2^ (Fe, GPR) = 0.72 which is similar to Lasso. By contrast, coefficients of determination for Mn vary strongly between the three methods: R^2^ (Mn, Lasso) = 0.55, R^2^ (Mn, PLSR) = 0.21 and R^2^ (Mn, GPR) = 0.13.

### 3.5. Aluminium

**Figure 6:**
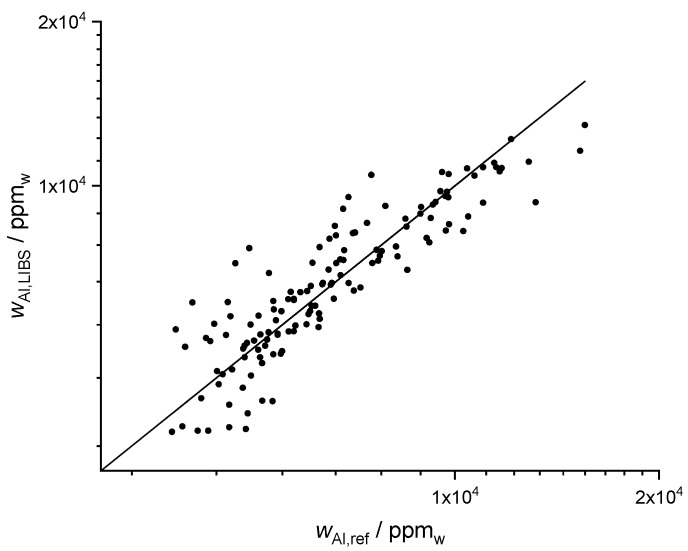
Results of 10-fold GPR cross validation for Al with R^2^ (Al, GPR) = 0.81.

Al was chosen as a constituent of most minerals in soils. Furthermore, it can induce root damage and plant growth reduction in acidic soils. The Al content of 6450 ppm in the investigated soils is the second highest of all elements investigated. Accordingly, the regression coefficients obtained with the three methods are similarly high as those of Ca and Mg with R^2^ (Al, PLSR) = 0.79, R^2^ (Al, Lasso) = 0.74 and R^2^ (Al, GPR) = 0.81 (Figure 6).

### 3.6. Plant Available (PA) Phosphorus

In addition to the total mass fractions of nutrients, the mass fractions of nutrients potentially available to plants are especially interesting for farmers. Such a prediction should not be possible on the basis of a univariate calibration and can only be based on a multivariate regression, which includes correlations to other elements. As an example, the plant available mass fraction of P was investigated in this work. The best correlation was obtained by GPR with R^2^ (P_pa_, GPR) = 0.35 (Figure S1 in Supplementary Materials), which is a first proof-of-principle for predicting plant available nutrient contents based on LIBS. Lasso, with R^2^ (P_pa_, Lasso) = 0.25, and PLSR, with R^2^ (P_pa_, PLSR) = 0.22, yielded slightly worse predictions. It is notable that the prediction of P available for plants was better than the prediction of the total P content.

### 3.7. Humus and pH

**Figure 7:**
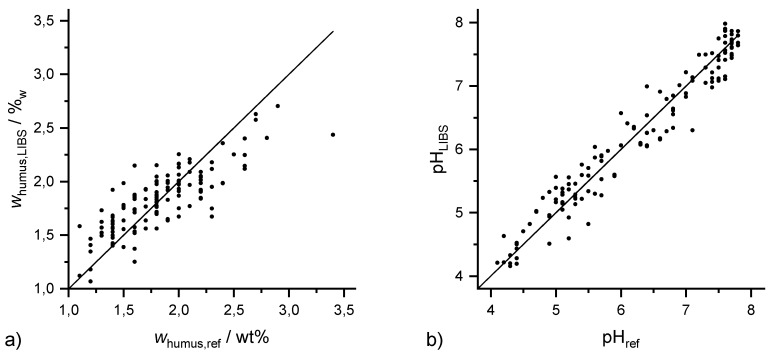
(**a**) Results of 10-fold Lasso cross validation for humus with R^2^ (humus, Lasso) = 0.66 and (**b**) 10-fold GPR cross validation for pH value with R^2^ (pH, GPR) = 0.95.

Finally, two important soil parameters, which are also interesting for farmers, are the humus content and the pH value. In Germany, humus denotes the total dead organic substance of the soil. The humus content can be estimated from the content of soil organic carbon by the simple equation humus = 1.72 × C_org_. Since this work is focused on precision agriculture, sample pretreatment for removal of inorganic carbonates in the samples analyzed with LIBS was not carried out. Therefore, a direct univariate calibration based on carbon lines can lead to erroneous results. However, multivariate calibration can consider the inorganic content by correlations with other elements, e.g., Ca or Mg. Multivariate regression of the Wilmersdorf samples yielded a good correlation (Figure 7a). The best prediction of humus was obtained by Lasso with R^2^ (humus, Lasso) = 0.66, followed by PLSR with R^2^ (humus, PLSR) = 0.56 and GPR with R^2^ (humus, GPR) = 0.54.

Soil pH is a measure for the proton activity in the soil solution. It is influenced by natural buffering due to clay minerals and organic matter. The pH value was measured in a soil suspension in a pH neutral buffer solution of CaCl_2_ with a pH meter. Similar to the measurement of the plant available phosphorus and the humus content, pH does not depend on a single element and cannot be determined by a univariate calibration. However, multivariate calibrations yielded successful predictions (Figure 7b) with R^2^ (pH, GPR) = 0.95 for GPR, R^2^ (pH, Lasso) = 0.92 for Lasso and R^2^ (pH, PLSR) = 0.91 for PLSR.

### 3.8. Interpretation of Lasso Coefficients

Lasso enables the simplest, most direct interpretation of the three multi-variate regression methods. Due to the strong tendency to shrink the regression coefficients of most of the predictor variables to zero, only predictor variables showing a good correlation with the dependent variable are included in the model. Non-zero coefficients indicate which emission lines were relevant for the regression. This is especially interesting for indirect correlations such as the prediction of plant available phosphorus.

**Figure 8:**
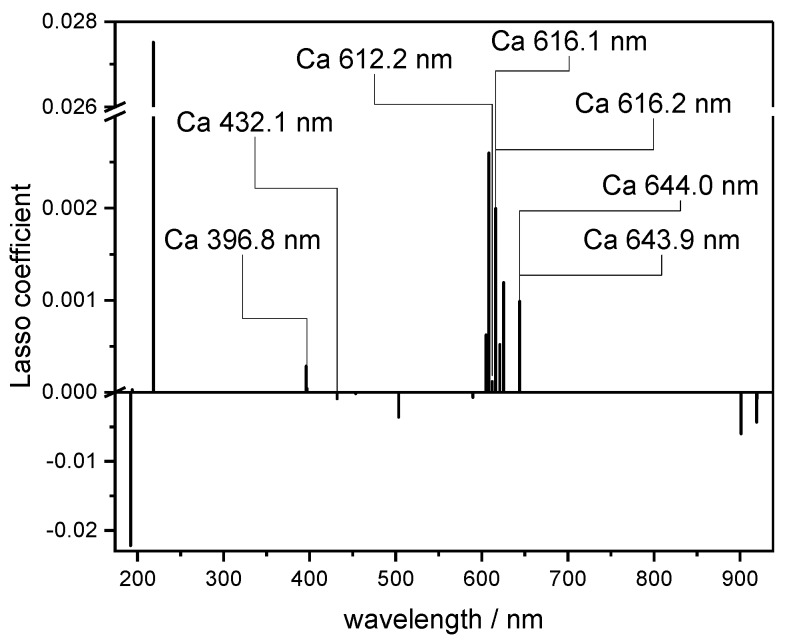
Lasso coefficients for Ca regression.

An example of coefficients obtained by Lasso for Ca is shown in Figure 8. The assignment of Lasso coefficients to the corresponding lines in the spectrum indicates that seven of fifteen coefficients are caused by Ca lines. If negatively correlated coefficients are neglected, the most positively correlated coefficients are matching the Ca lines. In the Lasso prediction models for N and P, the coefficients did not match the lines due to their low intensities. An inspection of the coefficients yields positive correlations to C lines, which can be explained by the fact that C and N are constituents of humus. Whereas in the case of humus, coefficients that can be assigned to C lines were found, in the case of pH, regression coefficients are found that can be assigned to different alkaline and alkaline earth elements (Na, K, Ca), which influence the pH value.

### 3.9. Comparison of PLS, Lasso and GPR

The coefficients of determination of the three multivariate methods are summarized in Table 2. While PLSR was selected as a standard method, which is very often used in LIBS, and more generally in spectroscopy literature, Lasso is also a robust method and allows a simple interpretation of regression coefficients (spectral lines). However, GPR is an interesting and more seldom applied non-parametric multivariate method in LIBS. As already discussed above, the coefficients of determination of multivariate methods depend on element concentrations, concentration ranges, line numbers and strengths, and matrix effects. Therefore, the best results were obtained for Ca, Mg, Fe and Al (higher concentration, number of lines and line strengths). The worst result was observed for P (low mass fraction, fewer and weak lines). The deviation in the coefficients of determination between the three methods is relatively small for larger coefficients (>0.5). For smaller coefficients (<0.5), larger deviations between the methods are observed. GPR yields the best results for six soil parameters, and Lasso yields the best results for three soil parameters. Lasso and GPR are especially more stable in the case of soil parameters which are difficult to predict. This applies to N, Mn and humus in the case of Lasso and the total and plant available P content in the case of GPR. In these cases, two properties of the two regression methods can be of importance, the robust selection of features in Lasso and the non-parametric regression in GPR.

Below, an investigation of several methods of data pretreatment with the aim of obtaining the best results with the multivariate methods and of finding a method of data treatment optimized for the application of handheld LIBS instrumentation in precision agriculture is reported.

### 3.10. Variance Reduction

Pořizka et al., [43] investigated a method of variance reduction prior to multivariate classification which is based on the Euclidean distance of a data point to the center of the coordinate space constructed by the first three principal components. Though this method did not yield the best results in the work cited, it is an interesting approach, which we also applied in the multivariate regression analysis. In this approach, the coefficients of determination of PLSR were compared in order to assess the effect of a removal of 5%, 20% and 50% of the spectra with the largest distance to the center of gravity in PCA. Subsequent to the removal of these spectra, all remaining spectra of one data point are averaged. The results obtained with the modified averaged spectra are compared to those containing the averages of all spectra. In contrast to the data pretreatment applied to all calculations shown above, the spectra were not background corrected and normalized before they were averaged. The results are shown in Table 3. The trends for the different elements are not uniform. Averages of the coefficients of determination in the columns of Table 3 are a measure for the general trend. Averaging after removal of 5% of the spectra yields the best results (largest average). However, the difference to averaging without removal of data is small. After further removal of spectra (20%, 50%), the coefficients of determination decrease. Although this was observed for most elements, a few elements (e.g., P) show an increase. The reason for the small decrease of the coefficients of determination is not clear. It could be simply a statistical effect related to a decreasing sample size. Despite these results, the application of this method of variance reduction could be useful, especially for difficult multivariate regression problems.

### 3.11. Data Pretreatment: Background-Correction and SNV-Normalization

Standard data pretreatment of real-world LIBS spectra currently consists of background correction and averaging in most cases, and normalization in many. In this work, the influence of background correction and normalization on the performance of the three multivariate methods was evaluated in relation to the averaged raw spectra. SNV was the normalization method used. The results are shown in Table 4.

Background correction and normalization improve the coefficients of determination of the multivariate regressions of the logarithmic and non-logarithmic Ca mass fractions. Especially the coefficients of determination of the regression with logarithmic mass fractions are improved. These are higher than the coefficients obtained by regression of non-logarithmic mass fractions. This is due to the better consideration of smaller mass fractions in the logarithmic regression, which do not significantly contribute to the non-logarithmic regression.

The coefficients of determination are increased for most elements (soil parameters) after applying background correction and normalization. The coefficients of determination of the different regression methods were averaged for all elements (soil parameters) in order to obtain better and more general trends and the relative increase was calculated. The enhancement is different for the three multivariate methods. Only a small increase of 6% was obtained for PLSR. The enhancement for GPR is 11% and the largest increase of 31% was observed for Lasso. The relatively small increase for PLSR (most common method) could explain why normalization is not applied more often in LIBS studies.

### 3.12. Data-Reduction

The amount of data collected in a measurement campaign of precision agriculture on a field is huge. Therefore, data reduction is an important topic. The approach chosen in this work is based on the integration of lines upon background correction. A threshold in the integration module based on the signal-to-noise ratio determines the number of lines considered and thus the extent of the data reduction. While a high threshold can decrease the number of lines to the most intense lines, a low threshold can include the noise of the baseline. In this work, five different thresholds were selected, which consider both extreme cases and three levels in between. While a plain text file which includes spectra of 137 data points has a size of 17,745 kB, the file after processing with the highest threshold has a size of only 35 kB.

The results of these investigations are summarized in Table 5 and refer to the mass fraction of Ca. All three methods produce similar results and GPR achieves the best performance followed by PLSR and Lasso. The strongest decrease of the performance of the multivariate methods is observed for the transition from raw or background corrected spectra to integrated spectra with the lowest signal-to-noise ratio (SNR). A plateau is reached for the subsequent SNR values and the coefficients of determination for the highest SNR value are much smaller. All multivariate methods show this behavior. It is remarkable that the performance remains nearly constant up to a SNR of 10, which corresponds to 115 data points per spectrum. Therefore, only a small loss of information occurs with increasing data reduction. This means that most of the information in the spectra is concentrated in the peaks clearly visible. On the other hand, the information found in the weak signals is required for maximum performance of the multivariate regression methods. For an assessment of the computational efficiency, all processes have to be carefully evaluated, which was beyond the scope of this work. A practical scenario could be the online implementation of the data reduction during the measurement campaign on board of the sensor platform, while the evaluation of the data could be performed offline at a later stage. This would result in a manageable amount of data and enable maximum flexibility in the choice of multivariate methods applied later.

## 4. Conclusion

A commercially available handheld LIBS spectrometer was used for a spatially resolved determination of nutrients and various soil parameters in two agricultural fields. Measurements were conducted in the laboratory. Univariate calibration methods do not usually allow the transfer of a calibration method obtained for one field to a second field. Multivariate methods, however, often better account for matrix effects and have the potential for creating more generalized calibration models. Three different multivariate regression methods (PLSR, Lasso, GPR) were characterized and compared for measuring soil parameters. Lasso and GPR yielded better regression results than PLSR. The focus of this work was the determination of several major and minor nutrients. While several nutrients, such as Ca, Mg, K and Fe, could be determined with good accuracy. Other nutrients, such as Mn and P, could only be determined qualitatively with the handheld instrument. The performance of the multivariate regression models depended on several factors such as the element concentration, the concentration range, the number of lines, the line strengths, and matrix effects. In addition to the mass fractions of nutrients in their elemental form, additional soil parameters were investigated. These include the plant available P content, the humus content and the soil pH. Several methods of data pretreatment, namely variance reduction, background correction and normalization, were tested. The latter method in particular has the potential of yielding improved multivariate regression results. Data reduction of the huge amounts of data generated during a measurement campaign can be performed without a significant loss of information in the multivariate regression.

## Figures and Tables

**Table 1:** Summary of the lines and the average mass fractions of the nutrients determined by reference analytics ICP-OES, signal-to-noise ratios of very weak lines in parentheses.

Nutrients	Observed Lines, λ/nm	Average Mass Fractions/ppm
Ca	315.9, 317.9, 370.6, 373.7, 393.3, 396.8, 422.7, 430.2, 443.5, 445.5, 518.9, 527.0, 551.4, 558.9, 585.8, 610.3, 612.2, 616.2, 643.9, 646.2, 649.4, 849.8 (3), 854.2	4950
K	404.6, 691.1 (2), 693.9 (4), 766.5, 769.9	1280
Mg	278.0, 279.5, 280.2, 285.2, 333.5	1450
N	746.8 (5), 821.6 (4), 868.3 (4)	917
P	213.6 (<2), 547.7 (<2)	372
Fe	193.6 (<2), 239.5, 248.8, 272.7 (3), 274.9, 301.8 (<2), 321.7 (2), 358.6 (4), 374.2, 405.5, 428.5, 438.4	10400
Mn	259.3, 279.8, 293.7, 294.8, 322.9 (4), 324.2, 344.1, 346.1, 403.3, 408.3, 476.3 (4), 478.4 (4), 482.4 (5)	249
C	193.1 (5), 247.8	
Al	220.8, 221.1, 226.4 (2), 226.9 (3), 236.7, 237.3, 256.8, 257.5, 265.2 (3), 266 (4), 308.2, 309.3, 394.4, 396.2	6450

**Table 2:** Comparison of coefficients of determination of PLSR, Lasso and GPR methods, number of Lasso coefficients (Min/1SE) in parenthesis.

Soil Parameter	PLSR	Lasso (Min/1SE)	GPR
Ca	0.87	0.85/0.83 (56/31)	0.89
Mg	0.79	0.75/0.69 (27/16)	0.78
K	0.64	0.65/0.59 (51/16)	0.66
N	0.51	0.65/0.60 (34/10)	0.51
P	0.14	0.21/0.18 (18/8)	0.28
Fe	0.77	0.76/0.71 (52/27)	0.72
Mn	0.21	0.55/0.51 (51/29)	0.13
Al	0.79	0.74/0.72 (76/36)	0.81
P (pa)	0.22	0.25/0.11 (57/10)	0.35
Humus	0.56	0.66/0.58 (47/10)	0.54
pH	0.91	0.92/0.91 (36/32)	0.95

**Table 3:** Effect of outlier elimination based on the Euclidean distance of a data point to the center of the principal component space, comparison of coefficients of determination for PLSR.

Soil Parameter	All Spectra	5% Removal	20% Removal	50% Removal
Ca	0.71	0.66	0.51	0.52
Mg	0.73	0.73	0.73	0.72
K	0.60	0.60	0.60	0.54
N	0.48	0.47	0.48	0.43
P	0.18	0.24	0.28	0.22
Fe	0.69	0.70	0.68	0.66
Mn	0.15	0.14	0.15	0.13
Al	0.72	0.73	0.72	0.69
P (pa)	0.25	0.27	0.30	0.24
Humus	0.58	0.63	0.55	0.58
pH	0.86	0.85	0.85	0.83
mean	0.54	0.55	0.53	0.51

**Table 4:** Effect of background correction and normalization of spectra on multivariate methods, reported as coefficients of determination, in the case of Ca, values in parentheses show the effect of using the logarithms of the mass fractions.

Element	Averaged Raw Spectra			Background Corrected, Normalized and Averaged Spectra		
	PLSR	Lasso	GPR	PLSR	Lasso	GPR
Ca	0.82 (0.68)	0.84 (0.59)	0.86 (0.82)	0.86 (0.87)	0.84 (0.85)	0.83 (0.89)
Mg	0.73	0.71	0.75	0.79	0.75	0.78
K	0.60	0.64	0.60	0.64	0.65	0.66
N	0.48	0.56	0.41	0.51	0.65	0.51
P	0.18	0.16	0.26	0.14	0.21	0.28
Fe	0.69	0.63	0.64	0.77	0.76	0.72
Mn	0.15	0.07	0.01	0.21	0.55	0.13
Al	0.72	0.65	0.71	0.79	0.74	0.81
P (pa)	0.25	0.09	0.37	0.22	0.25	0.35
Humus	0.58	0.41	0.50	0.56	0.66	0.54
pH	0.86	0.77	0.93	0.91	0.92	0.95
mean	0.52	0.47	0.52	0.55	0.61	0.57
change				6%	31%	11%

**Table 5:** Effect of data reduction for different signal-to-noise ratios (SNR) on coefficients of determination obtained for the Ca mass fraction for the three multivariate methods PLSR, Lasso and GPR, application to 137 spectra (samples).

Method	Raw	Background Corrected	SNR 1	SNR 3	SNR 5	SNR 10	SNR 22
File size/kB	17,745	7601	254	137	95	61	35
Data points/spectrum	7701	7701	238	179	149	115	81
R² (PLSR)	0.82	0.80	0.73	0.76	0.76	0.76	0.70
R² (Lasso)	0.84	0.80	0.79	0.80	0.82	0.74	0.64
R² (GPR)	0.86	0.85	0.79	0.73	0.75	0.83	0.76

## Supplementary Materials

The following are available online at https://www.mdpi.com/1424-8220/20/2/418/s1, Table S1: Comparison of RMSEP of PLSR, Lasso and GPR methods, Figure S1: Results of 10-fold GPR cross validation for plant available P, R2(P, GPR) = 0.35.
