# Supplementary material for: Soil Nutrient Detection for Precision Agriculture Using Handheld Laser-Induced Breakdown Spectroscopy (LIBS) and Multivariate Regression Methods (PLSR, Lasso and GPR)
Source: Sensors (Basel). 2020 Jan 11;20(2):418. doi: 10.3390/s20020418 (PMC7014682; doi:10.3390/s20020418)
Supplement: Supplementary file 1 [file sensors-20-00418-s001.pdf]

Supplementary Material

Soil Nutrient Detection for Precision Agriculture Using Handheld Laser-Induced Breakdown Spectroscopy (LIBS) and Multivariate Regression Methods (PLSR, Lasso and GPR)

Alexander Erler [1], Daniel Riebe [1], Toralf Beitz [1], Hans-Gerd Löhmannsröben [1,*] and Robin Gebbers [2]

[1] Physical Chemistry, University of Potsdam, Karl-Liebknecht-Str. 24-25, 14476 Potsdam, Germany; aerler@uni-potsdam.de (A.E.); riebe@uni-potsdam.de (D.R.); beitz@uni-potsdam.de (T.B.)

[2] Leibniz Institute for Agricultural Engineering and Bioeconomy (ATB), Max-Eyth-Allee 100, 14469 Potsdam, Germany

* Correspondence: loeh@chem.uni-potsdam.de

Received: 20 December 2019; Accepted: 9 January 2020; Published: date

In this supplementary material, the RMSEP of the different soil parameters are provided for the multi-variate methods PLSR, Lasso and GPR (Table S1). Table S1 supplements the coefficients of determination given in Table 2 of the publication.

Table S1. Comparison of RMSEP (in ppm, except for humus and pH) of PLSR, Lasso and GPR methods

Parameter	PLSR	LASSO	GPR
Ca	2560	2900	2570
Mg	311	334	313
K	245	258	254
N	162	137	162
P	80.4	76.9	73.7
Fe	1710	1760	1880
Mn	46.9	35.1	49.1
Zn	6.17	7.22	7.23
Al	1110	1220	1050
P (pa)	3.33	3.26	3.04
Humus	0.270	0.238	0.276
pH (Ca)	0.342	0.329	0.264

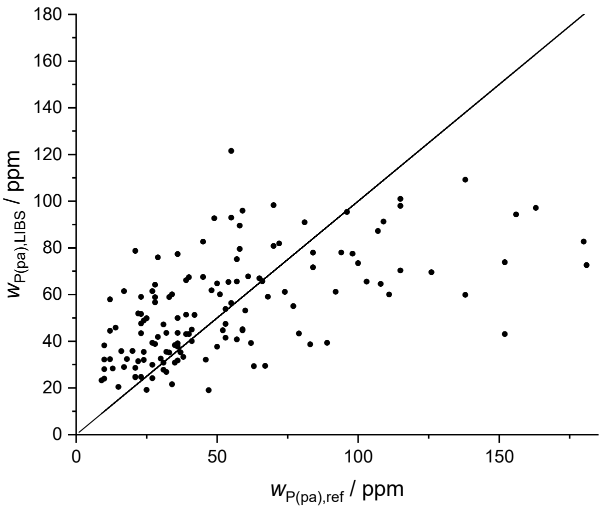

Figure S1. Results of 10-fold GPR cross validation for plant available P, R^2(P, GPR) = 0.35.
